# Circulating Tumour DNA in Patients With *EGFR* ‐Mutated Non‐Small‐Cell Lung Cancer and Early Disease Progression After First‐Line Osimertinib Treatment: The ELUCIDATOR Multicentre Prospective Observational Study

**DOI:** 10.1002/cam4.70861

**Published:** 2025-04-05

**Authors:** Akihiro Tamiya, Yasuyuki Mizumori, Mitsuo Osuga, Shun‐ichi Isa, Yoshihiko Taniguchi, Keiichi Nakamura, Daijiro Harada, Tsutomu Shinohara, Hidetoshi Yanai, Katsumi Nakatomi, Masahide Oki, Masahide Mori, Tomohito Kuwako, Koji Yamazaki, Atsuhisa Tamura, Masahiko Ando, Yasuhiro Koh

**Affiliations:** ^1^ Department of Internal Medicine National Hospital Organization Kinki‐Chuo Chest Medical Center Sakai Osaka Japan; ^2^ Department of Respiratory Medicine National Hospital Organization Himeji Medical Center Himeji Hyogo Japan; ^3^ Center for Biomedical Sciences Wakayama Medical University Wakayama Japan; ^4^ Clinical Research Center National Hospital Organization Kinki‐Chuo Chest Medical Center Sakai Osaka Japan; ^5^ Department of Respiratory Medicine National Hospital Organization Asahikawa Medical Center Asahikawa Hokkaido Japan; ^6^ Department of Thoracic Oncology and Medicine National Hospital Organization Shikoku Cancer Center Matsuyama Ehime Japan; ^7^ Department of Respiratory Medicine National Hospital Organization Kochi Hospital Kochi Japan; ^8^ Department of Respiratory Medicine National Hospital Organization Mito Medical Center Ibaraki Japan; ^9^ Department of Respiratory Medicine National Hospital Organization Ureshino Medical Center Ureshino Saga Japan; ^10^ Department of Respiratory Medicine National Hospital Organization Nagoya Medical Center Nagoya Aichi Japan; ^11^ Department of Thoracic Oncology National Hospital Organization Osaka Toneyama Medical Center Toyonaka Osaka Japan; ^12^ Department of Respiratory Medicine National Hospital Organization Shibukawa Medical Center Shibukawa Gunma Japan; ^13^ Department of Thoracic Surgery National Hospital Organization Kyushu Medical Center Fukuoka Kyushu Japan; ^14^ Department of Respiratory Medicine National Hospital Organization Tokyo National Hospital Tokyo Japan; ^15^ Department of Advanced Medicine and Clinical Research Nagoya University Hospital Nagoya Japan; ^16^ Internal Medicine III Wakayama Medical University Wakayama Japan

**Keywords:** circulating tumour DNA, EGFR mutations, next‐generation sequencing, non‐small‐cell lung cancer, osimertinib, resistance mechanisms

## Abstract

**Background:**

Osimertinib is the standard therapy for patients with chemotherapy‐naive advanced non‐small‐cell lung cancer (NSCLC) harbouring sensitising epidermal growth factor receptor (*EGFR*) mutations. However, some patients treated with osimertinib experience progressive disease (PD). Therefore, this study aimed to explore mechanisms underlying osimertinib resistance, focusing on early PD (within 6 months).

**Methods:**

This multicentre prospective observational study enrolled patients with advanced NSCLC receiving osimertinib as the first‐line anti‐cancer therapy. Mutations in cancer‐associated genes were analysed using next‐generation sequencing of circulating tumour DNA samples collected before osimertinib treatment and on detection of PD.

**Findings:**

Between May 2019 and January 2021, 188 patients were enrolled, of whom 125 (66%) were women and 96 (51%) had *EGFR* exon 19 deletion mutations. In this interim analysis, 78 patients experienced PD and 36 experienced early PD. Compared with patients without early PD, those with early PD were more likely to test positive for *EGFR*‐activating mutations at baseline (86.1% vs. 63.4%, *p* = 0.009) and had significantly more co‐occurring gene mutations in addition to *EGFR* mutations (2.89 ± 1.49 vs. 1.97 ± 1.37, *p* = 0.002). In three patients with early PD, one patient each had a germline *BRCA1*, *BRCA2* and *BRINP3* mutation.

**Conclusion:**

*EGFR* mutations in ctDNA and multiple co‐occurring gene mutations at baseline are associated with poor outcomes and early PD. Plasma‐based serial comprehensive gene profiling could help predict and identify patients who are unlikely to benefit from osimertinib treatment.

**Trial Registration:** Japanese Register of Clinical Trials JRCT: registration number: jRCTs031180051

**Abbreviations**
EGFR: epidermal growth factor receptor
JRCT: Japanese Register of Clinical Trials
MET: mesenchymal–epithelial transition
NGS: next‐generation sequencing
NSCLC: non‐small‐cell lung cancer
OS: overall survival
PFS: progression‐free survival
PS: performance status
RECIST: Response Evaluation Criteria in Solid Tumors
TKI: tyrosine kinase inhibitors
TNBC: triple‐negative breast cancer
VAF: variant allele frequency

## 1 Introduction

Lung cancer represents the leading cause of cancer‐related mortality globally, with non‐small‐cell lung cancer (NSCLC) constituting approximately 75% of all lung cancer cases [1]. Targeted therapies are actively being developed to improve treatment effectiveness in select patient populations [2], and epidermal growth factor receptor (*EGFR*) tyrosine kinase inhibitors (TKI) are the first choice for NSCLC harbouring *EGFR*‐sensitising mutations, given their superior effectiveness, even when immune checkpoint inhibitors (ICIs) are clinically introduced [3]. Furthermore, osimertinib is a third‐generation oral EGFR‐TKI that potently and selectively inhibits both *EGFR* harbouring *EGFR*‐sensitising mutations and *EGFR T790M* resistance mutations irreversibly [4]. Previous studies have reported that osimertinib is more effective than first‐generation EGFR‐TKIs, gefitinib or erlotinib, in promoting progression‐free survival (PFS) and overall survival (OS) [3, 5].

In the Phase III AURA3 study analysing circulating tumour DNA (ctDNA) samples, common resistance mechanisms included the *EGFR* C797S mutation and amplification of mesenchymal–epithelial transition (*MET*) and human epidermal growth factor receptor 2 (HER2) [6, 7, 8]. Meanwhile, the FLAURA study identified *MET* amplification and *EGFR* C797S mutation as the most frequent resistance mechanisms detected using ctDNA and next‐generation sequencing (NGS) [9]. Furthermore, some data from previous reports are available on the histological or molecular resistance mechanisms to first‐line osimertinib [10, 11]. However, the data on resistance mechanisms to first‐line osimertinib remain still unknown. Therefore, post‐treatment strategies for failed first‐line osimertinib have not yet been established, highlighting the need for further research investigating these mechanisms of resistance.

Furthermore, in the FLAURA study, approximately 15% of patients who received osimertinib experienced progressive disease (PD) within 6 months [5]. Factors contributing to early PD should be identified for determining initial treatment strategies and subsequent approaches after osimertinib use. In this first report from a prospective observational study (the ELUCIDATOR study), we aimed to explore candidate mechanisms and primary resistance factors associated with early progression, which we defined as occurring within 6 months of osimertinib administration. We analysed plasma samples collected at baseline and at PD points from patients with advanced NSCLC harbouring *EGFR*‐sensitising mutations and report on candidate resistance mechanisms, including the potential primary mechanisms, to first‐line osimertinib.

## 2 Methods

### 2.1 Study Design and Patients

The ELUCIDATOR study was designed as a prospective observational study and conducted at multiple centres of the National Hospital Organization Group in Japan. The details of the ELUCIDATOR study have already been described previously [12]. The purpose of the ELUCIDATOR study was to evaluate resistance‐related mutations of osimertinib as first‐line chemotherapy for advanced NSCLC harbouring EGFR‐sensitising mutations. Patient eligibility criteria included (1) a definitive diagnosis of non‐squamous NSCLC confirmed through biopsy or cytology, (2) *EGFR* mutations (exon 19 deletion or exon 21‐point mutation L858R), (3) osimertinib as the first‐line anti‐cancer therapy and (4) the ability to provide blood specimens. Written informed consent was obtained from all patients before the study was performed, and the central review board approved this protocol before the start of the study (central review board of CRB3180018; National Hospital Organization Review Board for Clinical Trial). This prospective observational study was registered in the Japanese Register of Clinical Trials (JRCT; registration number: jRCTs031180051).

The ctDNA analyses presented here were exploratory, prespecified and prospective analyses of patients treated with osimertinib as first‐line treatment. All patients meeting the eligibility criteria who were examined for plasma *EGFR* mutations using NGS at baseline were included. In this interim analysis, early PD was defined as PD within 6 months of osimertinib treatment.

### 2.2 Plasma Circulating Tumour DNA Analysis

Serial plasma samples were collected at baseline, 3 weeks, 12 weeks and on detection of PD. This analysis examined paired plasma samples collected at baseline and after PD, up to January 2023. Although this study allowed for osimertinib use after disease progression, samples from these patients were collected after PD occurred. If treatment discontinuation occurred during osimertinib administration, such as due to adverse events, patients were followed up for as long as possible without treatment, and samples were collected after PD. Plasma ctDNA samples were analysed using NGS with CAncer Personalised Profiling by deep Sequencing (CAPP‐Seq) technology‐based assay [13] (AVENIO ctDNA Surveillance Kit; Roche Diagnostics, Indianapolis, IN, USA). The assay targeted somatic mutations in the whole exon and hotspot regions of 197 cancer‐related genes, and allowed us to detect copy number variation (CNV). Variant calls were performed using AVENIO ctDNA Analysis Software (Roche Diagnostics, Indianapolis, IN, USA) that utilises Exome Aggregation Consortium (ExAC), the 1000 Genomes Project and the Single Nucleotide Polymorphism Database (dbSNP) with a limit of detection for the variant allelic fraction set at 0.1%. These variants were then deemed cancer‐related somatic mutations according to the Catalogue of Somatic Mutations in Cancer (COSMIC) and The Cancer Genome Atlas (TCGA). Synonymous mutations were excluded from the analyses, and ClinVar information was manually annotated for BRCA1/2 mutations (Table 2). Variants with a VAF ranging from 40% to 100% were classified as germline mutations unless aneuploidy could not be denied. In addition, CNV analysis by the AVENIO ctDNA analysis software provides the CNV score using both on‐target reads and nonspecifically captured off‐target reads to calculate log_2_ of copy ratios across the genome for each sample. We also excluded the samples with CNV score 0–5 regarded as putatively equivocal.

### 2.3 Assessments

Tumour response by computed tomography or magnetic resonance imaging was conducted at baseline (within 28 days of assignment) and when investigators consider CT imaging necessary (at least once every 3 months). PD was assessed according to the Response Evaluation Criteria in Solid Tumors (RECIST) version 1.1 [14]. Acquired mechanisms and primary mechanisms of resistance were identified by comparing paired plasma samples collected at baseline and on PD detection in patients with and without detectable plasma *EGFR* mutations. The during‐treatment period was defined as the time from administration of osimertinib until PD.

### 2.4 Statistical Analysis

In the present study, an exploratory analysis was performed, and data were summarised using descriptive statistics. The original statistical considerations of the ELUCIDATOR study have been described elsewhere [12]. Briefly, the main statistical analysis focused on resistance‐related gene mutations of osimertinib detected by NGS using ctDNA. Detection ratios and 95% confidence intervals, along with quantitative values of resistance‐related gene mutations of osimertinib, were calculated. PFS was defined as the time between osimertinib administration and confirmed disease progression or death. OS was defined as the time between osimertinib administration and death. Plasma samples at progression included in the paired analysis were collected up to December 2022. Clinical data were analysed using 31 December 2022 as the data cut‐off. Patients with events occurring after 31 December 2022 were censored in the analysis.

## 3 Results

### 3.1 Patient Characteristics

Between May 2019 and January 2021, 188 patients were enrolled in the ELUCIDATOR study and treated with osimertinib. Among these, 10 patients were excluded from the analysis due to withdrawal of consent during the study or failure to meet the inclusion criteria; plasma samples from 178 patients were evaluated for the clinical data (PFS and OS), and sufficient ctDNA samples from 167 samples were analysed using NGS. Among them, 78 patients, including 36 with early PD, experienced PD before the data cut‐off. Paired plasma samples were obtained from all 36 patients who experienced early PD (Figure S1). We focused on early PD patients in the present study; therefore, the final analysis of PD patients was described in another paper [15].

**FIGURE 1:**
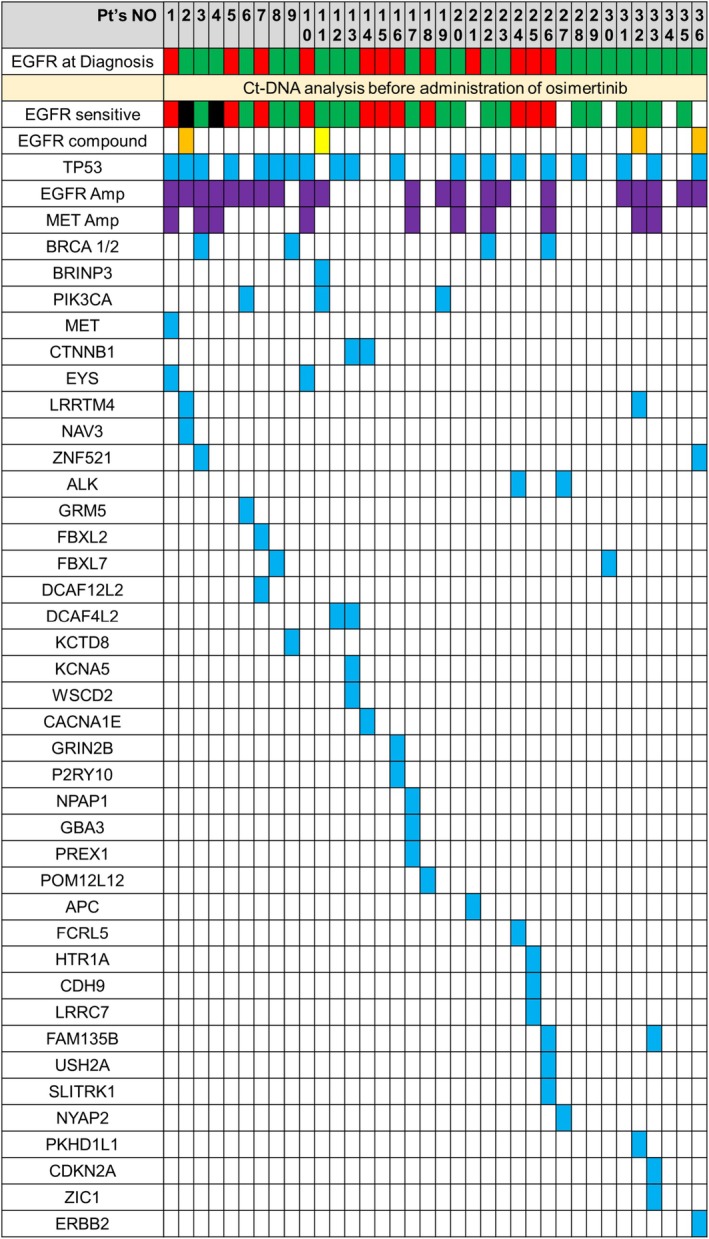
Mutational spectrum at baseline in patients with early PD. PD, progressive disease.

Among the 178 patients included in the analysis, the median age was 74 years, 125 (66%) were women, 151 (84%) had a performance status (PS) of 0 or 1, 90 (51%) had Stage IVB disease, 95 (53%) had an *EGFR* exon 19 deletion mutation and 104 (58%) had never smoked. Furthermore, among the 178 patients included in the analysis, 114 (64%) had *EGFR* mutations and 64 (36%) had *TP53* mutations detected in ctDNA. Further details on patient characteristics are shown in Table 1. Among the 36 patients who experienced early PD, the median age was 76.5 years, 20 (56%) were women, 29 (81%) had a PS of 0 or 1, 12 (33%) had an *EGFR* exon 19 deletion mutation and 15 (42%) had never smoked. Of the 36 patients, 31 (86%) had *EGFR* mutations and 19 (53%) had *TP53* mutations detected in ctDNA (Table 1). The mutational spectrum at baseline in the 36 patients with early PD is shown in Figure 1. At baseline, 21 (58%) patients had *EGFR* amplification and 10 (28%) had *MET* amplification. Four patients had EGFR compound mutations (L62R, T790M, A289T, G665S), four had *BRCA1/2* mutations, three had *PIK3CA* mutations, and one had a bone morphogenetic protein/retinoic acid inducible neural‐specific protein‐3 (*BRINP3*) mutation. Furthermore, OS was shorter among patients who experienced early PD than among patients without early PD (median OS 12.1 and 41.3 months, respectively; HR, 7.56; 95% CI, 4.35–13.12) (Figure S2).

**TABLE 1:** Patients' characteristics.

	Overall population (*n* = 178)	ctDNA analysis population (*n* = 167)	Early PD population (*n* = 36)
Age: median (range)	74 (36–91) years old	74 (36–91) years old	76.5 (48–88) years
Gender: male/female	59/119	56/111	16/20
PS: 0/1/2/3/unknown	67/84/21/4/2	61/82/20/4/0	8/21/6/1/0
Smoking: Current/former/never	8/66/104	8/62/97	1/20/15
Stage: IIIC/IVA/IVB	13/75/90	12/71/84	1/9/26
Brain metastasis: +/−	48/130	44/123	12/24
Liver metastasis: +/−	17/171	17/150	8/28
Bone metastasis: +/−	55/123	53/114	13/21
EGFR: del19+/L858R	95/83	91/76	12/24
EGFR from ct‐DNA: del19+/L858R/both/none/NE	54/55/5/53/11	54/55/5/53/—	11/18/2/5/0
TP53 from ct‐DNA: Positive/negative/NE	64/103/11	64/103/—	19/17/0
The number of all mutated genes, including del19+ and L858R: ≥ 3/< 3/NE	63/104/11	63/104/—	20/16/0

Abbreviations: ctDNA, circulating tumour DNA; EGFR, epidermal growth factor receptor; NA, not available; PD, progressive disease; PS, performance status.

### 3.2 Changes in the *EGFR* Variant Allele Frequency Between Baseline and Detection of Progressive Disease

Figure S3a shows changes in the *EGFR* variant allele frequency (VAF) between baseline and PD detection. In this study, we analysed paired ctDNA samples at baseline and PD detection. Therefore, if we could not analyse both ctDNA samples by NGS, we excluded these patients from the analysis of changes in the *EGFR* VAF. The proportion of patients with the *EGFR* VAF not so changed from baseline at PD was higher for patients with early PD than for patients who experienced PD after more than 6 months (Figure S3b,c).

### 3.3 Effect of Detection of *EGFR* Mutations at Baseline on PFS and OS

In the early PD group, 86.1% of patients had *EGFR* mutation alleles detected in ctDNA, and in the other patients, 60.3% had *EGFR* mutations detected in ctDNA (Figure S4a). The patients with early PD had a higher percentage of detectable ctDNA at baseline as compared to the remaining patients (*p* = 0.004). Furthermore, patients with *EGFR* mutations in ctDNA had shorter PFS and OS than those without *EGFR* mutations (median PFS: 13.5 vs. 24.4 months; HR, 2.30; 95% CI, 1.47–3.74), and the median OS was 26.9 months and NR, respectively (HR, 2.37; 95% CI, 1.27–4.84) (Figure S4b).

### 3.4 Number of Genetic Mutations in Patients With and Without Early Disease Progression

**FIGURE 2:**
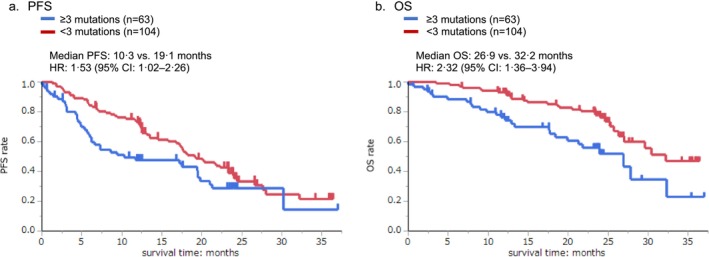
Kaplan–Meier curve of progression‐free survival (PFS) and overall survival (OS) according to the number of gene mutations. We classified the number of gene mutations including *EGFR* mutations as ≥ 3 mutations (*n* = 63) or < 3 mutations (*n* = 104). (a) Kaplan–Meier curve of PFS. (b) Kaplan–Meier curve of OS. CI, confidence interval; EGFR, epidermal growth factor receptor; HR, hazard ratio.

Patients with early PD had significantly more co‐occurring gene mutations combined with *EGFR* mutations compared with other patients (2.89 ± 1.49 vs. 1.97 ± 1.37, *p* = 0.002) (Figure S5). Patients with early PD also had more gene mutations overall, regardless of the detection of *EGFR* mutations. Furthermore, patients with three or more co‐occurring gene mutations had shorter PFS and OS than those with fewer than three gene mutations. The median PFS was 10.3 and 19.1 months, respectively (HR, 1.53; 95% CI, 1.02–2.26) (Figure 2a), and the median OS was 26.9 and 32.2 months, respectively (HR, 2.32; 95% CI, 1.36–3.94) (Figure 2b).

### 3.5 Relationship Between Germline Mutations and Early Disease Progression

Three of 36 patients with early PD and eight of 78 patients with PD exhibited a significant reduction in plasma *EGFR*‐mutant VAF at PD compared with that at baseline (Figure S3b,c). In these patients, the *EGFR* VAF was reduced by more than 10%, or the VAF was below the detection limit at PD. All three patients with early PD showed a significant reduction in plasma *EGFR*‐mutant VAF at the time of PD detection, and one patient each had germline *BRCA1*, *BRCA2* and *BRINP3* mutations (Table 2). Both the BRCA1 Q934* and BRCA2 R2318* mutations detected in this study were classified as pathogenic. The patient who had a *BRCA1* mutation was a 55‐year‐old man who was an ex‐smoker, and the patient who had a *BRCA2* mutation was a 77‐year‐old woman who had never smoked. Both these patients had brain metastases. Among the five patients with PD, excluding patients with early PD, one had a *TP53* germline mutation (Table 2). In this patient, although the *EGFR* VAF and VAF of the other gene mutations were markedly reduced at the time of PD detection, the allele frequencies of the *TP53* genes also remained unchanged.

**TABLE 2:** Early PD patients who harboured germline mutations with significant VAF reduction of EGFR post therapy.

Sample No.	Baseline			At the time of PD			PFS (months)
	Gene	AA Mut	VAF (%)	Gene	AA Mut	VAF (%)	
1	EGFR	L858R	18.699	EGFR	L858R	0.236	1.0
	BRCA1	Q934*	58.252	BRCA1	Q934*	49.377	
	TP53	V173M	17.209	TP53	V173M	0	
	ZNF521	A820V	0.145	ZNF521	A820V	0	
2	EGFR	L858R	2.348	EGFR	L858R	0.521	2.9
	BRCA2	R2318*	47.606	BRCA2	R2318*	47.919	
	TP53	A161T	1.289	TP53	A161T	0.729	
	KCTD8	R391H	0.956	KCTD8	R391H	0.432	
	FBXL7	P266S	0	FBXL7	P266S	0.411	
	PIK3CA	E81K	0	PIK3CA	E81K	0.280	
3	EGFR	L858R	38.042	EGFR	L858R	0.220	5.6
	EGFR	T790M	31.898	EGFR	T790M	0.177	
	BRINP3	E597K	45.428	BRINP3	E597K	49.490	
	PIK3CA	E542Q	11.189	PIK3CA	E542Q	0.116	
4	EGFR	E746_S752 > A	15.994	EGFR	E746_S752 > A	0	17.3
	TP53	C242Y	59.750	TP53	C242Y	45.955	

Abbreviations: AA, amino acid; PD, progressive disease; PFS, progression‐free survival; VAF, variant allele frequency. * indicates that the mutation results in a stop codon that terminates protein translation.

## 4 Discussion

This prespecified interim analysis of the ELUCIDATOR study data provides a characterisation of factors associated with early PD among patients with NSCLC treated with first‐line osimertinib. There are no standard criteria for defining early PD among patients treated with osimertinib. In the FLAURA study, approximately 15% of patients experienced PD within 6 months; therefore, we decided to define early PD as PD within 6 months. In fact, OS in early PD cases was much shorter than in non‐early PD cases (Figure S2) (median OS 12.1 and 41.3 months, respectively; HR, 7.56; 95% CI, 4.35–13.12). In this study, the median PFS and OS in the overall population were 19.1 (95% CI: 13.5–22.2) months and 36.0 (95% CI: 30.4–not reached [NR]) months [15], and this PFS and OS was consistent with the results of the FLAURA study [3, 5].

The results of the ctDNA analysis using NGS showed that the prevalence of *EGFR* mutations at baseline was higher in the early PD group than in the group without early PD and that the *EGFR* VAF was not decreased in most patients with early PD. Furthermore, patients with early PD had significantly more co‐occurring gene mutations than the other patients, and those with early PD had a much shorter OS than patients without early PD.

Previous reports have identified several baseline alterations associated with poor response to EGFR‐TKIs, including missense *TP53* mutations and *MET* amplifications [16, 17]. We did not analyse whether specific genes are associated with a reduced risk of early PD but found that *EGFR* amplification, *MET* amplification and co‐expressed genes, including *TP53*, were highly prevalent. Moreover, the presence of multiple co‐occurring mutations was associated with shorter PFS and OS. In the evolution of tumours, initial founder (clonal or trunk) somatic mutational events that drive tumorigenesis are manifested as clonal mutations. Genome‐doubling events frequently occur early in tumour evolution, situated at the trunk of the evolutionary tree; meanwhile, sub‐clonal driver events may subsequently arise following genome doubling within the branches of the tumour evolutionary tree [18]. Zhang et al. [19] applied multi‐region, whole‐exome sequencing to specimens obtained from 11 patients diagnosed with early‐stage lung adenocarcinoma and found an association between the number of sub‐clones in a tumour and the risk of relapse. They showed that a larger sub‐clonal mutation fraction may be associated with a higher risk of postsurgical relapse in patients with localised lung adenocarcinomas. In addition, a prospective observational study, which analysed surgical tissue specimens using NGS, found that a larger number of coexisting mutations was associated with shorter relapse‐free survival and OS, suggesting that multiple mutations are an indicator of cancer aggressiveness [20]. Furthermore, Blakely et al. [20] performed genomic analysis of ctDNA samples of patients with *EGFR*‐mutated lung cancer and demonstrated that the critical co‐occurring oncogenic events occurred in most advanced‐stage *EGFR*‐mutated lung cancers. Although osimertinib was used as a second‐line treatment, they compared ctDNA of osimertinib responders with that of osimertinib non‐responders and found that osimertinib non‐responders had a larger number of co‐occurring mutations than osimertinib responders and that cell‐cycle alterations were especially prevalent among non‐responders. In patients treated with osimertinib as first‐ or second‐line treatment, the presence of co‐occurring mutations in the early stage has been reported to be associated with a poor treatment response and shorter OS [21].

In this study, the ctDNA analysis showed that the prevalence of *EGFR* mutations at baseline was higher, and the *EGFR* VAF did not decrease in most patients with early PD. In previous reports, analysis of ctDNA at baseline in patients with *T790M* mutations treated with osimertinib as second‐line treatment showed that patients with low levels of *EGFR*‐sensitising mutations in ctDNA (VAF < 6.1%) had better PFS and OS [22]. Compared with patients without *EGFR* mutations, those with detectable *EGFR* mutations in plasma had worse outcomes [22]. In another study of patients with *EGFR*‐mutated NSCLC treated with EGFR‐TKI, patients with *EGFR* mutations at baseline had shorter OS than patients without *EGFR* mutations at baseline (HR, 1.65; 95% CI, 0.90–3.02) [23]. Furthermore, in patients with *EGFR* mutations in plasma at baseline, the rate of clearing of *EGFR* mutations during the first 8 weeks of treatment was predictive of the clinical outcome [15]. Although the number of patients enrolled in these previous studies was small, the poorer response in patients with *EGFR* mutations and predictive value of the clearing rate of *EGFR* mutations are consistent with the findings of this study.

In this study, most patients with early PD had *EGFR* mutations at baseline, and the *EGFR* VAF did not significantly decrease at PD. However, three patients with early PD had a significant decrease in the *EGFR* VAF between baseline and PD detection. In these patients, only the VAFs of the germline gene mutations, including *BRCA1/2* and *BRINP3*, did not change between baseline and PD detection. The VAFs of all the other co‐occurring gene mutations, including *EGFR*, were markedly decreased. To the best of our knowledge, this is the first report suggesting that germline gene mutations are associated with the poor effectiveness of EGFR‐TKIs in NSCLC harbouring *EGFR*‐sensitising mutations. Five patients harboured BRCA1/2 mutations at baseline in the study cohort, and four experienced early PD. One patient who did not develop early PD harboured the BRCA2 V2503I mutation classified as a variant with uncertain significance (VUS) according to ClinVar. Though the involvement of germline BRCA mutations in EGFR‐TKIs resistance has not been previously reported, there is a literature that reports the potential association of the development of resistance to the ALK inhibitor lorlatinib and germline BRCA mutation in ALK‐rearranged lung adenocarcinoma patient and combined treatment of lorlatinib plus the PARP inhibitor olaparib was effective [24]. The mechanisms of developing resistance with the coexistence of germline mutations still remain elusive, and we believe more studies, including basic research, are warranted. There are some possible explanations for the poor response to osimertinib treatment observed in patients with germline gene mutations. First, triple‐negative breast cancer (TNBC), which is the most aggressive breast cancer subtype, disproportionately affects carriers of *BRCA* mutation [25]. Activation of the EGFR‐KRAS‐SIAH pathway is a major tumour driver in chemo‐resistant TNBC [26]. Persistent high expression of SIAH in residual tumours following neoadjuvant chemotherapy or neoadjuvant systemic therapy suggests that the EGFR‐KRAS pathway remains activated, indicating an ineffective treatment response. In two patients with coexisting *EGFR* and *BRCA* mutations in this study, osimertinib inactivated the *EGFR* mutation pathway; however, wild‐type *EGFR* was activated, suggesting that osimertinib was not effective and did not prevent early PD. In addition, the involvement of *BRCA1* has been suggested in other biological processes such as apoptosis [27]. Apoptosis serves a critical function in the development of cancer, and the anti‐apoptotic inhibitor of apoptosis (IAP) family serves a pivotal function in this process [28]. Elevated IAP protein levels, specifically the X‐linked inhibitor of apoptosis (XIAP), cIAP1 and cIAP2, are prevalent in numerous varieties of cancer, contributing to the resistance of cancer cells to apoptosis [29]. XIAP expression has been reported to be particularly high in cancers with *BRCA1* mutations [30]. Therefore, because XIAP inhibits apoptosis, it is possible that apoptosis was not induced by the EGFR‐TKI. Second, *BRINP* family proteins suppress cell‐cycle progression [31], and *BRINP3* is a novel tumour suppressor gene in tongue squamous cell carcinoma [32]. Additionally, BRINP3 is a mitochondrial‐associated protein, which has been associated with tumour proliferation, migration and invasion [33]. In addition, in osteosarcoma, *BRINP3* has been associated with histological grade, tumour recurrence and poor clinical prognosis [34]. Furthermore, whole‐exome sequencing of specimens from patients with small‐cell lung cancer undergoing preoperative chemotherapy has revealed that *BRINP3* mutations may be associated with chemo‐resistance [35]. However, the reason for the association between *BRINP3* and tumour progression is unclear. The results of this study suggest that *BRINP3* is associated with a poor prognosis, indicating the need to explore reasons for the association between *BRINP3* and early PD.

This study has some limitations. First, plasma NGS analysis was limited to identifying genomic alterations detectable in ctDNA; therefore, non‐genetic resistance mechanisms were not evaluated, such as histological transformation, coexisting different histological tissues or alteration in protein expression. For instance, the transformation from lung adenocarcinoma to small‐cell lung cancer could not be pathologically confirmed [36, 37]. Second, we did not compare the mutations in tissue and plasma in this analysis. Although liquid biopsy provides valuable insights for monitoring and identifying emerging resistance mechanisms, these results highlight the necessity of complementary tissue testing for complete histological diagnosis. Third, although we analysed ctDNA using ultrasensitive NGS with a detection sensitivity of more than 0.1%, some patients did not have detectable ctDNA content in their plasma. PD can occur after the tumour has shrunk following osimertinib treatment, and ctDNA may not be detectable in the plasma due to the small tumour volume. Therefore, this analysis is descriptive, and additional resistance mechanisms were not identified. Finally, the number of participants in this study was too small to determine the mechanism of genetic mutation. Therefore, genetic analysis studies with a larger number of participants are needed to verify these results.

## 5 Conclusion

In patients with NSCLC, *EGFR* mutations in ctDNA and multiple co‐occurring gene mutations at baseline are associated with poor outcomes and early PD. In addition, germline mutations in BRCA1/2 and BRINP3 at baseline were found in patients with early resistance to osimertinib monotherapy, and it is warranted to further examine early PD events. Plasma‐based serial comprehensive gene profiling may be useful for identifying patients who are unlikely to benefit from osimertinib treatment; however, further studies are warranted.

## Author Contributions

**Akihiro Tamiya:** conceptualization (lead), data curation (equal), formal analysis (equal), funding acquisition (lead), investigation (lead), methodology (equal), project administration (equal), resources (equal), software (equal), visualization (equal), writing – original draft (lead), writing – review and editing (lead). **Yasuyuki Mizumori:** project administration (equal), supervision (supporting), writing – review and editing (supporting). **Mitsuo Osuga:** data curation (equal), formal analysis (equal), investigation (equal), methodology (equal), resources (equal), software (equal), validation (equal), writing – original draft (supporting), writing – review and editing (supporting). **Shun‐ichi Isa:** conceptualization (equal), data curation (equal), funding acquisition (supporting), methodology (equal), software (equal), supervision (supporting). **Yoshihiko Taniguchi:** conceptualization (equal), project administration (equal), resources (equal), supervision (equal). **Keiichi Nakamura:** project administration (equal), supervision (equal). **Daijiro Harada:** project administration (equal), supervision (equal). **Tsutomu Shinohara:** project administration (equal), supervision (equal). **Hidetoshi Yanai:** project administration (equal), supervision (equal). **Katsumi Nakatomi:** project administration (equal), supervision (equal). **Masahide Oki:** project administration (equal), supervision (equal). **Masahide Mori:** project administration (equal), supervision (equal). **Tomohito Kuwako:** project administration (equal), supervision (equal). **Koji Yamazaki:** project administration (equal), supervision (equal). **Atsuhisa Tamura:** project administration (equal), supervision (equal). **Masahiko Ando:** conceptualization (supporting), data curation (supporting), formal analysis (supporting), methodology (supporting), resources (equal), software (equal), supervision (equal), visualization (equal). **Yasuhiro Koh:** conceptualization (equal), data curation (lead), formal analysis (lead), funding acquisition (equal), investigation (equal), methodology (lead), resources (equal), software (equal), supervision (equal), validation (equal), writing – original draft (supporting).

## Conflicts of Interest

Akihiro Tamiya received honoraria from Eli Lilly, Ono Pharmaceutical, Chugai Pharmaceutical, Boehringer Ingelheim, AstraZeneca, Bristol‐Myers Squibb, Amgen, Taiho Pharmaceutical, Kyowa Kirin, MSD, Takeda Pharmaceutical, Nihon‐Kayaku, Novartis, Thermo Fischer, Amgen, Tsumura, Daiichi‐Sankyo and Merck BioFarma, and research funding from Daiichi‐Sankyo, Beigene and AstraZeneca. Yoshihiko Taniguchi received honoraria from Chugai Pharmaceutical, Ono Pharmaceutical, AstraZeneca and MSD. Daijiro Harada received honoraria from Takeda Pharmaceutical, Eli Lilly, Chugai Pharmaceutical, AstraZeneca, Taiho Pharmaceutical, Ono Pharmaceutical, Bristol‐Myers Squibb, Towa Pharmaceutical and Boehringer Ingelheim. Masahide Oki received honoraria from AMCO, AstraZeneca, Canon Medical Systems, Chugai Pharmaceutical, Fujifilm Toyama Chemical, Kaneka Medix, Merit Medical Japan, Novartis Pharma, Olympus and Sanofi, and research funding from AbbVie, AstraZeneca, Chugai Pharmaceutical, Fujifilm Toyama Chemical, GlaxoSmithKline, Janssen Pharmaceutical, MSD, Ono Pharmaceutical, Parxel International, Pfizer and Sanofi. Masahide Mori received honoraria from AstraZeneca, Boehringer Ingelheim, MSD, Eli Lilly, Novartis, Chugai Pharmaceutical, Taiho Pharmaceutical, Kyowa‐Kirin, Ono Pharmaceutical, Otsuka, Nihon‐Kayaku, Pfizer, Daiichi‐Sankyo, Takeda Pharmaceutical and Shionogi, and research funding from Chugai Pharmaceutical, Ono Pharmaceutical, MSD and Delt‐fly. Yasuhiro Koh received honoraria from Chugai Pharmaceutical, Guardant Health, Amgen, Takeda Pharmaceutical and Tosoh Corporation, and had a consulting or advisory role at Tosoh Corporation and received research funding from Boehringer Ingelheim, AstraZeneca, Chugai Pharmaceutical, Tosoh Corporation, Daiichi Sankyo, Zeon Corporation, Amgen and Takeda Pharmaceutical. Other co‐authors received no honoraria and research funding.

## Supporting information


Data S1.

## Data Availability

The datasets used and/or analysed during the current study are available from the corresponding author upon reasonable request.
