# Supplementary material for: Circulating Tumour DNA in Patients With EGFR ‐Mutated Non‐Small‐Cell Lung Cancer and Early Disease Progression After First‐Line Osimertinib Treatment: The ELUCIDATOR Multicentre Prospective Observational Study
Source: Cancer Med. 2025 Apr 5;14(7):e70861. doi: 10.1002/cam4.70861 (PMC11971570; doi:10.1002/cam4.70861)
Supplement: Supplementary file 1 — Data S1. [file CAM4-14-e70861-s001.docx]

**Supplementary material**

**Circulating tumour DNA in patients with *EGFR*-mutated non-small cell lung cancer and early disease progression after osimertinib treatment: The ELUCIDATOR multicentre prospective observational study**

Tamiya et al.

| **Contents** | **Page** |
| --- | --- |
| Supplementary figure 1. Flow diagram of patient selection | 2 |
| Supplementary figure 2. Kaplan–Meier curve of overall survival (OS) in patients with and without early disease progression | 3 |
| Supplementary figure 3. Change in the *EGFR* variant allele frequency between baseline and PD detection | 4 |
| Supplementary figure 4. Effect of *EGFR* mutation detection at baseline on tumour response, progression-free survival (PFS), and overall survival (OS) | 5 |
| Supplementary figure 5. Difference in the number of mutated genes in patients with early disease progression (PD) and other patients | 7 |

**
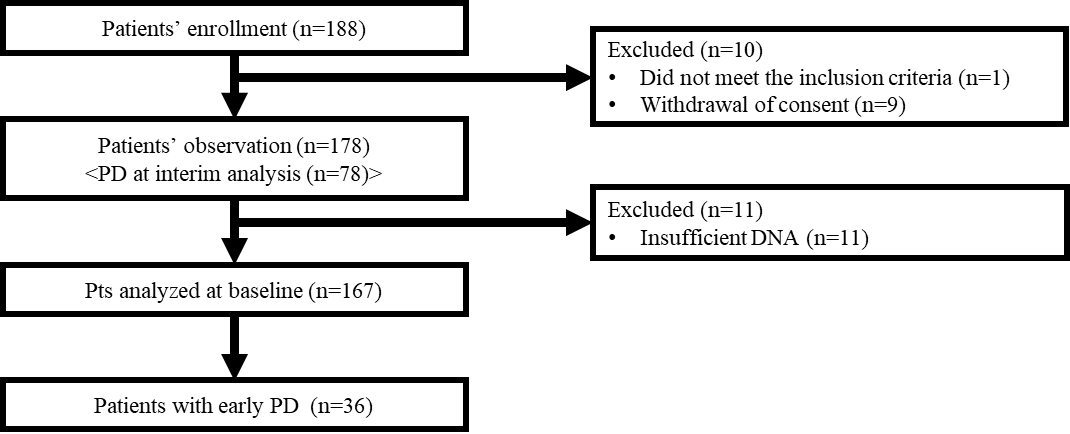
**

**Supplementary figure 1. Flow diagram of patient selection.** In total, 188 patients were enrolled in the ELUCIDATOR study between May 2019 and January 2021

PD: progressive disease.

| 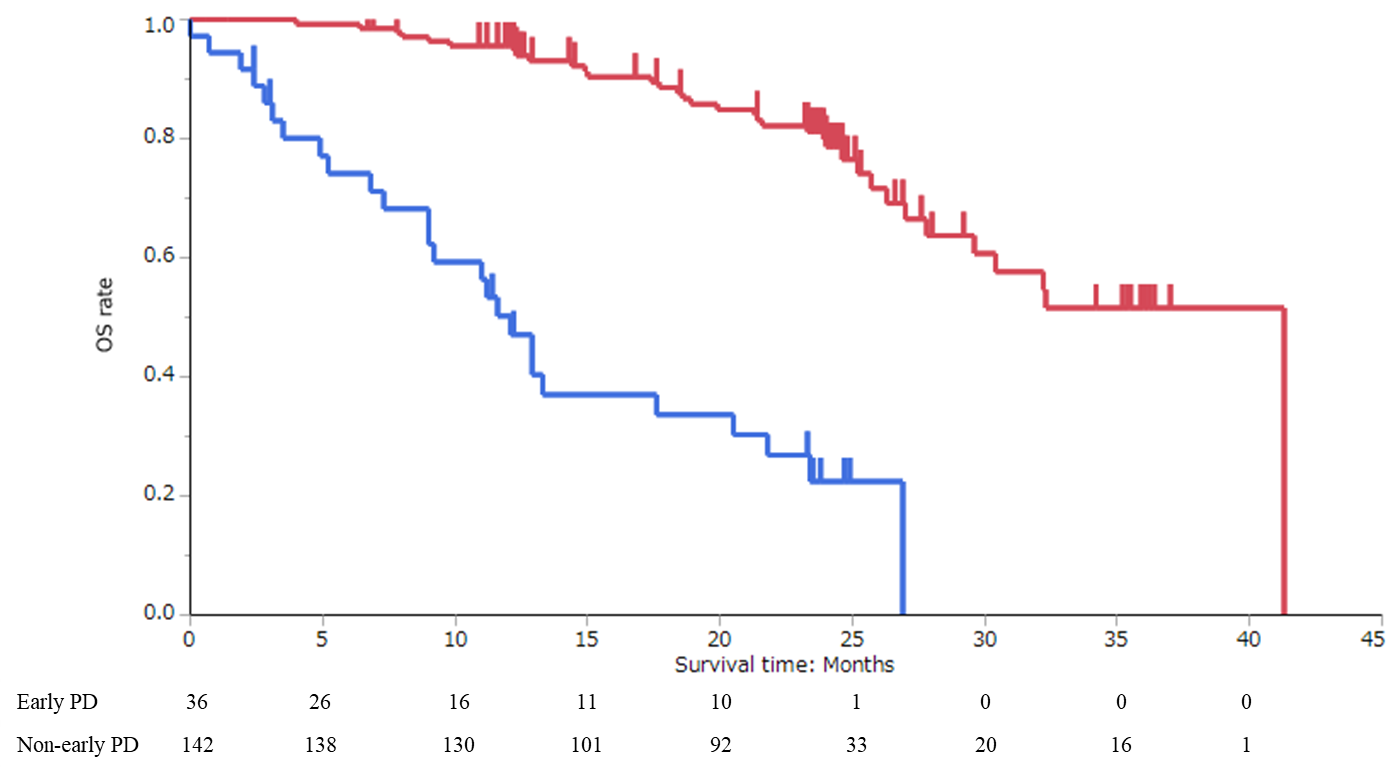 | Patients with early PD (n=36)  Patients without early PD (n=142)  Median OS: 12·1 vs. 41·3 months  HR: 7·56 (95% CI: 4·35–13·12) |
| --- | --- |

**Supplementary figure 2. Kaplan–Meier curve of overall survival (OS) in patients with and without early disease progression**

PD: progressive disease; OS: overall survival; HR: hazard ratio; CI: confidence interval.

1. b. c.

| 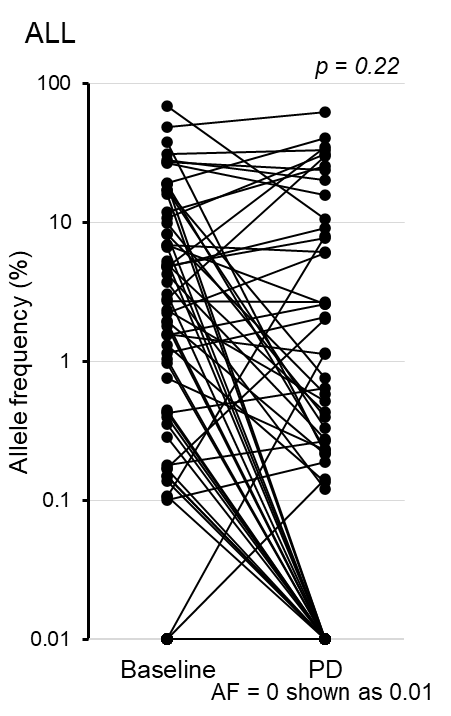 | 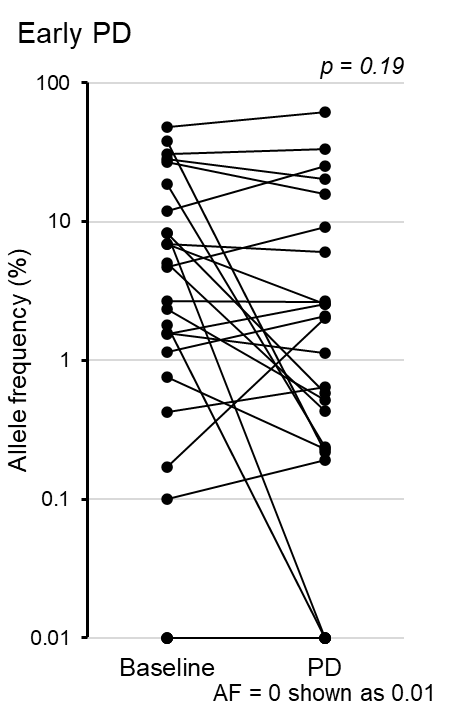 | 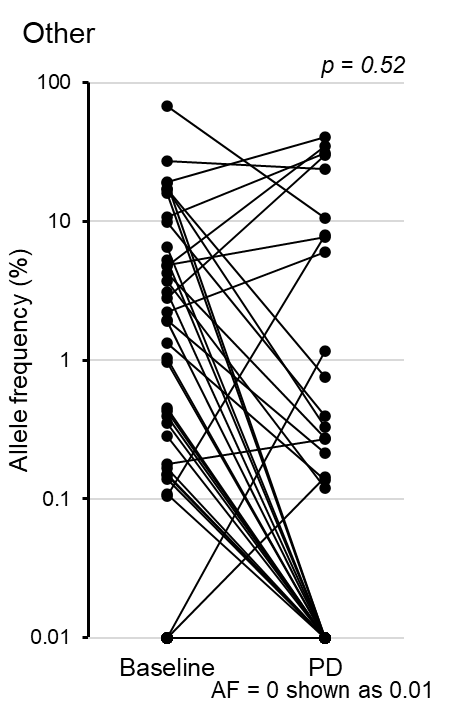 |
| --- | --- | --- |

**Supplemental figure 3. Change in the *EGFR* variant allele frequency between baseline and PD detection**

ALL: all patients with PD. Early PD: Patients who experienced PD within 6 months of osimertinib treatment. Other: Patients who experienced PD after 6 months. AF: allele frequency; PD: progressive disease.

| a.  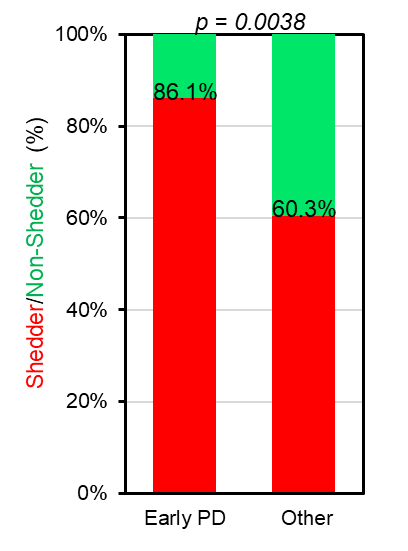 | b. PFS |  |
| --- | --- | --- |
|  | 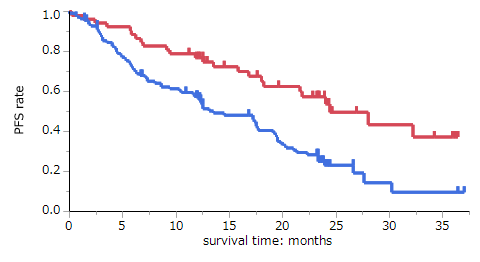 | Median PFS: 13·5 vs. 24·4 months  HR: 2·30 (95% CI: 1·47–3·74)  With *EGFR* mutations (n = 114)  Without *EGFR* mutations (n = 53) |
|  | c. OS |  |
|  | 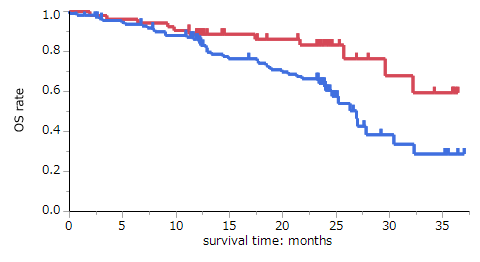 | Median OS: 26·9 vs. NR months  HR: 2·37 (95% CI: 1·27–4·84)  With *EGFR* mutations (n = 114)  Without *EGFR* mutations (n = 53) |

**Supplemental figure 4. Effect of *EGFR* mutation detection at baseline on tumour response, progression-free survival (PFS), and overall survival (OS)**

*EGFR* mutations were detected in circulating tumour DNA (ctDNA) at baseline. No *EGFR* mutations were detected by ctDNA at baseline. Patients with *EGFR* mutations (n=114) and patients without *EGFR* mutations (n=53) are represented. (a) Comparison of the proportion of patients with *EGFR* mutations in patients with early disease progression (PD) and patients without early PD. (b) Kaplan–Meier curve of PFS. (c) Kaplan–Meier curve of OS.

HR: hazard ratio; CI: confidence interval; PD: progressive disease; EGFR: epidermal growth factor receptor; NR, not reached

| a   | b  **** | c   |
| --- | --- | --- |

**Supplementary figure 5. Difference in the number of mutated genes in patients with early disease progression (PD) and other patients**

(a) The number of mutated genes, including del19+ and L858R, in all participants. (b) The number of mutated genes, excluding del19+ and L858R, in all participants. (c) The number of mutated genes, excluding del19+ and L858R in participants with *EGFR* mutations.

PD: progressive disease; EGFR: epidermal growth factor receptor.
